# Amyloid Beta and Tau Proteins as Therapeutic Targets for Alzheimer's Disease Treatment: Rethinking the Current Strategy

**DOI:** 10.1155/2012/630182

**Published:** 2012-03-08

**Authors:** Siddhartha Mondragón-Rodríguez, George Perry, Xiongwei Zhu, Jannic Boehm

**Affiliations:** ^1^Le Groupe de Recherche sur le Système Nerveux Central, Département de Physiologie, Université de Montréal, Montréal, QC, Canada; ^2^Douglas Mental Health University Institute, McGill University, Department of Psychiatry, Montreal, Quebec, Canada; ^3^UTSA Neurosciences Institute and Department of Biology, College of Sciences, University of Texas at San Antonio, San Antonio, TX, USA; ^4^Department of Pathology, Case Western Reserve University, Cleveland, OH, USA

## Abstract

Alzheimer's disease (AD) is defined by the concurrence of accumulation of abnormal aggregates composed of two proteins: Amyloid beta (A**β**) and tau, and of cellular changes including neurite degeneration and loss of neurons and cognitive functions. Based on their strong association with disease, genetically and pathologically, it is not surprising that there has been a focus towards developing therapies against the aggregated structures. Unfortunately, current therapies have but mild benefit. With this in mind we will focus on the relationship of synaptic plasticity with A**β** and tau protein and their role as potential targets for the development of therapeutic drugs. Finally, we will provide perspectives in developing a multifactorial strategy for AD treatment.

## 1. Introduction

**Amyloid Beta: The Therapeutic Strategy**

Alzheimer's disease (AD) is histopathologically characterized by extraneuronal amyloid-beta protein (A*β*) deposits. Historically, A*β* has been related to cell toxicity and genetic evidence provides the basis for its proposal as the primary cause of the disease [1, 2]. Supporting these hypotheses, impairment in A*β* clearance by the central nervous system (CNS) has been reported in AD patients [3]. In cultured neurons, A*β* was shown to activate apoptotic pathways, leading to caspase activation, ultimately contributing to neurodegeneration [4]. It was also found that A*β* can activate apoptosis signal-regulating kinase (ASK1) that is required for ROS- and ER-stress-induced JNK activation and apoptosis, mainly through production of reactive oxygen species (ROS), but not through endoplasmic-reticulum-(ER-) mediated stress [2, 5]. A*β* has been found to modulate redox factor-1 that plays crucial roles in both cell death signaling pathways and DNA repair by interacting with transcription factors such as AP-1, NF-kappaB, and p53 and directly participating in the cleavage of apurinic/apyrimidinic DNA lesions, therefore affecting both the cell death signaling pathways and DNA repair [6]. Furthermore, A*β* was responsible for inducing oxidative stress, predominantly via mitochondria, which also affected cholesterol balance [7] and can cause neurotoxicity due to production of free radicals [8].

The proposed mechanism for A*β* to exert the neurotoxic effects was the assembly into A*β* plaques and oligomers [9]. This was further nurtured by the finding of a genetic component associated to the hypothesis of A*β* deposition. Mutations in the amyloid precursor protein (APP) that facilitate its cleavage to generate amyloid peptide and/or mutations in presenilin-1 (PS-1) or presenilin-2 (PS-2), that promote amyloid peptide formation and consequently A*β* deposition have also been reported [10–12]. Although the genetic component is documented, we have to mention that far less than 1% of the worldwide AD cases are based on APP or PS mutations. Indeed, in a systematic genetic study of AD patients in a Latin American population, PS and APP mutations were absent [13].

A*β*-containing plaques have been classified into subtypes, such as senile, diffuse, and neuritic [14], with diffuse plaques having little impact on cognitive function, whereas neuritic plaques are associated with cognitive decline [15]. Therefore, it was hypothesized that diffuse plaques appear during early preclinical stages of the disease with later appearance of neuritic plaques. However the “peptide to plaque” model remains debated [16]. In recent years, other stages of A*β* aggregation, beside plaques, seem to play a role in the development of AD [17]. In this regard it has been shown that the A*β* aggregation/oligomerization process is playing a central role in pathogenesis; in other words, one soluble A*β* molecule (monomer) interacts with other A*β* monomers to form dimers, oligomers, and polymers, each state of aggregations forming a potentially pathogenic entity. Indeed, different states of soluble aggregates have been strongly related to synaptic loss and cognitive impairment [18], a topic that we will discuss later. From these data, the aggregation state (i.e., the different oligomers) seems to be related to the disease. Given that, disaggregation of a plaque will lead to an increase of A*β* monomers/oligomers and therefore could cause neurotoxicity. Furthermore, inhibiting the formation of aggregates will preserve monomeric or polymeric structures and, therefore, could cause neurotoxicity. Overall, the perceived strength of the amyloid cascade hypothesis is reflected in the scientific literature, which is voluminous and dominated by experimental studies that adhere to the following statement: A*β* accumulates in the AD brain, consequently leading to neurodegeneration [19]. Although spatial distribution of increased levels of A*β* is related to AD pathology [20] and some degree of correlation with neuronal loss has been reported [21], no strong clinical correlation between plaque deposition and the degree of cognitive decline during AD has been found [22]. It is therefore in question whether the A*β* plaque is responsible for all the damage seen during the process of neurodegeneration or not.

**An Alternative Point of View**

We have suggested that A*β* deposition as plaques could represent the effect rather than the cause of AD [23, 24]. A*β* aggregation may not be a harbinger of death, but rather a protective response to neuronal insult [25]. And, perhaps most contrary to current thinking, due to the fact that there is a negative correlation between A*β* deposition and oxidative damage [26], it has been proposed that diffuse amyloid plaques may be a compensatory response aimed at reducing oxidative stress [27–30]. If this hypothesis is correct, it means that A*β* deposits are simply a compensatory response, explaining the failure of therapeutic approaches simply directed to removal of A*β* plaques [31].

## 2. A***β***: The Therapeutic Target

Presently A*β* is one of the main therapeutic targets. In fact, immunization with A*β* was successful at removing A*β* from the brain. Imaging studies in AD patients showed that immunization with A*β* decreased amyloid plaques in the brain; however, this had no effect on cognition [32]. In mouse models, immunization has cleared small deposits and diffuses A*β* surrounding fibrillar cores [33]. But beyond removing A*β*, immunization has failed to clearly improve cognition in patients [34]. Active vaccination with A*β* in patients with mild-to-moderate AD in a phase II trial showed CNS inflammatory response [35], that was blamed for the failure. However, the inflammatory response has not been definitively proven to be the cause for the failure. Despite this outcome, new vaccines are presently in trial: Eli Lilly's solanezumab and Janssen and Pfizer's bapineuzumab (originally developed by the Dublin-based company Elan), are using monoclonal antibodies that work with the immune system, binding to amyloid-*β* and helping to clear accumulated amyloid-*β* peptides in the brain. Both are being tested in phase III trials on thousands of participants with mild-to-moderate Alzheimer's disease [36]. Following the hypothesis of preventing aggregates, *β*-secretase and *γ*-secretase inhibitors have also been used therapeutically, but with mixed results due to inhibition of other vital pathways, for example, the Notch pathway [37]. Although the main goal is to block the production of A*β* [37], *γ*-secretase inhibitors also block the proteolytic processing and function of Notch, which is essential for brain morphogenesis, making *γ*-secretase a worthwhile but difficult target for intervention [38]. In sum, the therapeutic outcome of current A*β*-related trials is rather discouraging. While there are without a doubt multiple factors that could explain a negative outcome of these trials, the above hypothesis raises the possibility that A*β* plaques are not a viable primary therapeutic target. Overall, there is little debate about A*β* being involved in AD pathology, but a strong debate about whether it is an appropriate therapeutic target.

## 3. Tau Protein: The Therapeutic Approach

Tau is an axonal protein that regulates microtubule stability [39]. According to current AD hypotheses, (a) tau becomes abnormally phosphorylated, (b) dissociates from microtubules and, (c) aggregates into neurofibrillary tangles (NFTs) [40, 41]. Tau has at least 45 phosphorylation sites, most of them are located in the proline-rich region (P-region) (residues 172–251) and the C-terminal tail region (C-region) (residues 368–441) [42]. Tau phosphorylation at both of these regions affects its capacity to interact with microtubules [43]. In terms of AD development, the phosphorylation sites located in the C-terminal region seem to play an interesting role. Phosphorylation at Ser262 selectively impairs binding of tau to microtubules [44]. Phosphorylation at Ser202 enhances tau polymerization; phosphorylation at the two neighbouring sites Ser202-Thr205 makes filament formation more sensitive to small changes in tau concentration [45]. Taking these data together, it seems that multiple phosphorylation events of tau, rather than just singular phosphorylation, play a crucial role during AD-related tau pathology [46, 47]. 

The abnormal phosphorylation of tau during AD is either related to an increase in kinase activity (glycogen synthase kinase 3*β*, cyclin-dependent kinase-5, p42/44 MAP kinase, p38 MAPK, stress-activated protein kinases, mitotic protein kinases) and/or a decrease in phosphatase activity (protein phosphatases 1, 2a, 2b) [48–52]. Certainly one kinase known to be of importance to tau phosphorylation is GSK3*β* [53]. In terms of phosphatase deregulation, it has been shown that protein phosphatases PP1, PP2A, PP2B, and PP5 dephosphorylate tau in vitro at sites Ser199, Ser202, Thr205, Thr212, Ser214, Ser235, Ser262, Ser396, Ser404, and Ser409 [54]. Of all phosphatases, PP2A was found to be the strongest tau-related phosphatase [54].

## 4. Therapeutic Strategy

 Tau phosphorylation is established as a major factor during AD with a therapeutic focus on its kinases and phosphatases [55, 56]. Recently, it was reported that sodium selenate was able to reduce tau phosphorylation by stabilizing PP2A, therefore, mitigating tau pathology in transgenic AD models [57]. Targeting PP2B, several neuroleptics (such as chlorpromazine, trifluoperazine, and clozapine) have also been suggested for AD treatment [58]. In an approach, parallel to prevent A*β* plaque formation, drugs that prevent tau aggregation have also been developed [59, 60]. At the end, the answer is quite the same: no proven success has yet emerged from these approaches.

### 4.1. Synaptic Plasticity: The Link

#### 4.1.1. Synaptic Plasticity and Kinases

Synaptic plasticity has been proposed to play a central role in brain capacity to incorporate transient experiences into persistent memory traces. Synaptic transmission can be enhanced (long-term potentiation, LTP) or depressed (long-term depression, LTD) by activity, and these changes can range from seconds to hours and days [61, 62]. Importantly, the affected intracellular pathways leading to LTP or LTD activation involve several kinases, such as GSK3*β*, SRC family tyrosine kinases, protein kinase A, protein kinase C, and, in particular, Ca^2+^/calmodulin-dependent protein kinase II [63, 64], which are known to play a role in AD. Given the importance of synaptic plasticity, it is not surprising that these phenomena could be affected during neurodegeneration and AD [65]. Presently, there is growing evidence that A*β* and tau are involved in synaptic dysfunction [66, 67]. Reports had placed A*β* close to synaptic terminals [68]. Indeed, A*β* was found to enhance N-methyl-D-aspartate (NMDA) receptor function by direct interaction [69]. A*β* was also found responsible for changes in presynaptic mechanism at the CA1–CA3 synapse of pyramidal neurons in the hippocampus [70] and for functional deficits in the mossy fibre pathway [71]. Furthermore, A*β* leads to decreased mitochondria in dendrites that resulted in the reduction in the number of spines or synapses [72, 73] and has been related to neuritic degeneration [74]. In the context of synaptic plasticity, A*β* leads to impairment of LTP [75] and facilitates LTD [76, 77]. Of note, A*β* has been suggested to exacerbate synaptic mitochondrial alterations including increased oxidative stress, decreased respiration, and compromised calcium handling capacity [78], all of them having an impact on synaptic plasticity. Overall, there is growing evidence showing that A*β* is related to changes in synaptic function.

#### 4.1.2. Phosphorylation as the Link

The critical question to raise at this point is how A*β* and tau are interconnected during the disease. Although the relationship between these two proteins remains vague, data has lent support to the hypothesis that phosphorylation of tau protein could be the key linking mechanism. Ten years ago it was found that A*β* fibrils accelerate the formation of abnormally phosphorylated neurofibrillary tangles (NFTs) in a tau transgenic mouse [79]. The following years, it was reported that A*β* could induce tau phosphorylation and toxicity in cultured septal cholinergic neurons [80]. More recently, it has been shown that A*β* oligomers cause abnormal tau phosphorylation and morphology changes of spines by missorting of endogenous tau into dendrites [81]. Finally, natural A*β* isolated from AD brains is sufficient to induce AD-type tau phosphorylation and, consequently, neuritic dystrophy [74]. Concerning synaptic plasticity, it has been found that the absence of tau protein inhibits the impairment of LTP and neurotoxicity caused by A*β* [82] and targeting tau by immunotherapy prevents cognitive decline in a tangle mouse model [83]. Additionally, recent data show that tau protein has a dendritic function in targeting the Src kinase Fyn to postsynaptic NMDA receptors [84]. Summarizing these data, it appears that synaptic failure and neurotoxicity induced by A*β* require tau phosphorylation. Not surprisingly, elimination of tau has been suggested as a therapeutic target in order to ameliorate disease progression [85]. However, neuronal alterations that underlie symptoms of AD are not exclusively due to a direct toxic effect of A*β* [24]. Furthermore, we do not know the molecular mechanisms underlying the complex changes of synaptic plasticity during AD. Just recently, we have found that tau protein has a physiological function at the synaptic terminal that is regulated by tau phosphorylation (unpublished data). Therefore, simply getting rid of tau protein as some have suggested could adversely affect the equilibrium of different forms of synaptic plasticity. The question remains: is synaptic plasticity the link between A*β* and tau? Clearly, the potency to look at AD from a synaptic perspective is that it integrates A*β* and tau in a functional concept.

## 5. Conclusion and Perspectives

Pathogenesis of AD comprises neurodegeneration in the hippocampal area of brain that is critically involved in learning and memory. Presently, synaptic plasticity (LTP and LTD), the process by which synapses modulate their connections with other neurons, seems to be playing an important role in response to injury and disease. But more importantly, emerging evidence suggests that synaptic dysfunction beside neuronal death is leading to cognitive failure associated with AD. In fact, parts of AD pathogenesis could be explained by a loss of synaptic plasticity. In this regard, a growing amount of data is showing that A*β* and tau protein are both necessary to cause changes in synaptic plasticity. However, the question remains: should either A*β* or tau be the therapeutic targets? The concept that aggregation of A*β* and tau is deleterious to cells and amenable to therapeutic molecules may be too simplistic. Instead, upstream and downstream targets have to emerge as therapeutic options. In this context, synaptic plasticity modulators could be an interesting target. The most important aspect of this approach is that synaptic plasticity links A*β* and tau to the synaptic terminal. In this regard, some molecules have already been tested. Memantine, for example, is a partial antagonist of NMDA-receptor function, approved for moderate-to-severe Alzheimer's disease (AD) treatment within the USA and Europe (under the brand name Namenda (Forest), Axura and Akatinol (Merz), and Ebixa and Abixa (Lundbeck)), has some promise [86, 87]. Memantine, in its current therapeutic form, only slows down the neuronal degeneration process, but does not improve cognitive function [88]. However, Memantine might be beneficial to modulate the NMDA-receptor response in earlier stages of AD, where synaptic plasticity rather than neurotoxicity is playing a role. Furthermore, another target for therapeutic intervention would be Fyn kinase, a member of the Src kinase family, which is intricately involved with potentiating NMDA-receptor-dependent transmission [89]. Fyn kinase is receptive to changes in intracellular tau and extracellular A*β* at the synaptic terminal [84, 90]. Therefore, drugs that directly regulate Fyn activity might be beneficial. Like Fyn, there are other targets critically involved during synaptic plasticity and AD, as previously mentioned. One such target is GSK3*β*, which is necessary for the induction of synaptic LTD, while at the same time inhibits LTP [91].

**Figure 1:**
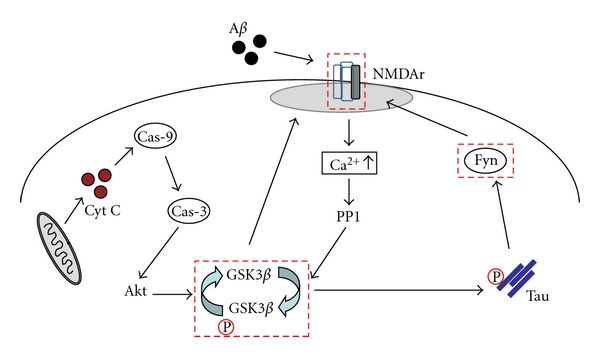
The role of GSK3*β* and Fyn during AD-related neurodegeneration and memory formation, along with NMDA receptor, makes them important therapeutic targets (red square). Impairment of hippocampal LTP by A*β* is through direct interaction with NMDA receptor. Calcium (Ca^2+^) enters via NMDA receptors and this leads to activation of protein phosphatase 1 (PP1), a key enzyme in synaptically induced LTD. PP1 can dephosphorylate GSK3*β* that determines whether NMDA receptor activation induces LTD or inhibits LTD. A*β* leads to decreased mitochondria and oxidative injury that promotes the release of cytochrome C (Cyt C) that may activate caspase-9 and caspase-3, which can cleave Akt, resulting in GSK3*β* activation. GSK3*β* under the control of Akt and PP1, is a critical determinant of the direction of NMDA receptor-dependent plasticity. The active GSK3*β* isoforms critically contribute to neurodegeneration by hyperphosphorylation of tau which deregulates Fyn activity and consequently affects NMDA receptor response.

In summary, it appears that the balance in synaptic plasticity during AD is tipped toward the induction of LTD. Hence, drugs that enhance LTP in combination with drugs that reduce induction of LTD might be of great value to treat AD. This therapeutic approach (i.e., simultaneously targeting critical check points for synaptic plasticity) will hopefully improve memory formation during AD (Figure 1). In sum, it is becoming all the clearer that approaches that focus on removing the pathological manifestations of AD might miss the intended outcome. Therefore, working with the biology of AD offers new hope for effective therapeutics.

## Abbreviations

AD: Alzheimer's disease
A*β*: Amyloid-beta protein
CNS: Central nervous system
ROS: Reactive oxygen species
APP: Amyloid precursor protein
ER: Endoplasmic reticulum
PS-1: Presenilin-1, or
PS-2: Presenilin-2
NFTs: Neurofibrillary tangles
LTP: Long-term potentiation
LTD: Long-term depression
PP1: Protein phosphatase 1
NMDA: N-methyl-D-aspartate receptor
